# Potential Anti-Skin Aging Effect of (-)-Catechin Isolated from the Root Bark of *Ulmus davidiana* var. *japonica* in Tumor Necrosis Factor-α-Stimulated Normal Human Dermal Fibroblasts

**DOI:** 10.3390/antiox9100981

**Published:** 2020-10-13

**Authors:** Sullim Lee, Jae Sik Yu, Hung Manh Phung, Jeong Gun Lee, Ki Hyun Kim, Ki Sung Kang

**Affiliations:** 1 Department of Life Science, College of Bio-Nano Technology, Gachon University, Seongnam 13120, Korea; sullimlee@gachon.ac.kr; 2 School of Pharmacy, Sungkyunkwan University, Suwon 16419, Korea; jsyu@bu.edu; 3 College of Korean Medicine, Gachon University, Seongnam 13120, Korea; 201940218@gc.gachon.ac.kr; 4 S-Skin Co., Ltd., #220, 17, Daehak 4-ro, Suwon 16226, Korea; bio.lee@s-skin.com

**Keywords:** skin aging, (-)-catechin, human dermal fibroblasts, tumor necrosis factor-α, reactive oxygen species

## Abstract

Reactive oxygen species (ROS) are generated during skin aging, including intrinsic (chronologic aging) and extrinsic aging (photoaging). Therefore, antioxidants that inhibit ROS generation can delay skin aging. In this study, we evaluated the potential anti-skin aging effect of (-)-phenolic compounds isolated from the root bark of *Ulmus davidiana* var. *japonica*. We preferentially investigated the possible preventive effects of isolates against the degradation of skin extracellular matrix. Among the isolates, (-)-catechin suppressed the activity of collagenase MMP-1, and reversed the degradation of collagen induced by tumor necrosis factor-α (TNF-α) in normal human dermal fibroblast. This action mechanism of (-)-catechin was validated by the suppression of tumor necrosis factor-α-induced accumulation of ROS and activation of mitogen-activated protein kinases, protein kinase B (Akt), and cyclooxygenase-2 (COX-2). The proinflammatory cytokines upregulate inflammatory reactions, and ultimately promote aging-related reactions. In this milieu, we demonstrated that (-)-catechin decreased the expression and secretion of proinflammatory cytokines, including interleukin (IL)-1β and IL-6. In conclusion, (-)-catechin is a candidate to ameliorate both intrinsic and extrinsic skin aging.

## 1. Introduction

Intracellular reactive oxygen species (ROS) are the main cause of various diseases in humans [1,2]. They are produced by oxidative phosphorylation in the mitochondria, and harmful foreign substances stimulate ROS production [3]. Generally, the human body produces antioxidants as a defense mechanism to eliminate most of the intracellular ROS. However, excess ROS can induce oxidative stress [4], which directly or indirectly causes DNA denaturation and cell membrane destruction, leading to aging, cancer, arteriosclerosis, diabetes, and neurodegeneration [5,6].

Aging-related changes occur in all organs of the human body. As the skin is in direct contact with the environment, skin aging is rapid as a result of environmental damage [7]. Skin aging can be divided into intrinsic aging (chronologic aging) and extrinsic aging (photoaging) [8]. Intrinsic aging is the natural aging process induced by reduced skin cell activity by ROS generated during metabolism in skin cells [9]. Extrinsic aging is caused by external environmental factors such as ultraviolet irradiation and pollutants [10]. The major causes of extrinsic aging are ROS-mediated secondary reactions that occur when UV rays are absorbed by the skin [11]. Excess ROS result in wrinkle formation via the cleavage and abnormal chain crossing of fibrous proteins such as collagen, elastic fibers, and glycosaminoglycans in the skin extracellular matrix. [4]. Collagen synthesis is inhibited by collagenases, including matrix metalloproteinase-1 (MMP-1) [12]. Therefore, to delay skin aging, it is important to identify antioxidants that suppress the production of ROS. During recent decades, antioxidants have been isolated from natural products.

*Ulmus davidiana* var. *japonica* (Rehder) Nakai (Ulmaceae) is widely distributed in various parts of Asia [13]. The stem and root barks of this species—yugeunpi in Korean traditional medicine—are known for their therapeutic potential against various diseases such as gastroenteric disorders, inflammatory disorders, edema, hemorrhoids, and jaundice [14,15,16,17]. Studies have demonstrated the pharmacological properties of *U. davidiana* extract, including antiarthritis effect [18], antiangiogenic activity [19], and immunocompetence-enhancing effect, by regulating inflammatory proteins [20]. Previously, we aimed to identify bioactive products from diverse natural sources [21,22,23,24]. We conducted a phytochemical investigation of the root bark of *U. davidiana* [25] and identified a new chromane derivative and 22 known compounds. These compounds were four catechin derivatives, one megastigmane glycoside, two dihydrochalcone glycosides, two flavanone glycosides, two coumarins, five lignan derivatives, and six phenolic compounds. We evaluated their potential biological activities, including neuroprotective, antineuroinflammatory, and anti-*Helicobacter pylori* activities [25].

In the present study, we investigated the root bark extract of *U. davidiana* using a liquid chromatography–mass spectrometry (LC/MS)-based phytochemical analysis method and isolated three phenolic compounds (**1**–**3**). The structure of these compounds was determined by interpreting one-dimensional (1D) and two-dimensional (2D) nuclear magnetic resonance (NMR) spectroscopic and LC/MS data, and the absolute configurations were established by comparing electronic circular dichroism (ECD) data and specific rotation. Generally, phenolic compounds such as flavonoids, as antioxidants, have the potential to suppress ROS generation. Thus, we evaluated the anti-skin aging effect of phenolic compounds from the root bark of *U. davidiana* in normal human dermal fibroblasts (NHDFs) to identify bioactive compounds. Herein, we describe the isolation and structure elucidation of compounds **1**, and their anti-skin aging effects in tumor necrosis factor-α (TNF-α)-stimulated NHDFs, and elucidate the action mechanism of the active compound (-)-catechin.

## 2. Materials and Methods

### 2.1. Plant Material

*U. davidiana* root barks were acquired in 2016 from Jecheon-si, Chungcheongbuk province, Korea, which was purchased from Donggwang General Corporation. The material was authenticated by one of authors, K. H. Kim. A voucher specimen (SKKU-NR 0401) of the material was stored at the herbarium in the School of Pharmacy, Sungkyunkwan University, Suwon-si, Korea.

### 2.2. Extraction and Isolation

*U. davidiana* root barks (5 kg) were dried and finely ground and extracted with 50% aqueous ethanol (EtOH) (60 L each × 2 days) under reflux and filtered. The filtrates were collected and concentrated under reduced pressure to afford crude EtOH extract (450 g). A portion of the extract (200 g) was suspended in distilled water (800 mL) to be solvent-partitioned with four solvents, including hexane, dichloromethane (CH_2_Cl_2_), ethyl acetate (EtOAc), and *n*-butanol (BuOH). This procedure resulted in the four main fractions of different polarities: hexane-soluble (2.5 g), CH_2_Cl_2_-soluble (25.0 g), EtOAc-soluble (18.0 g), and BuOH-soluble fractions (62.0 g). On the basis of the LC/MS results, the EtOAc fraction was determined for phytochemical investigation to identify the phenolic compounds.

The EtOAc fraction (18.0 g) was employed to chromatography on a Diaion HP-20 column in a MeOH solvent system (100% H_2_O, 20% MeOH-H_2_O, 40% MeOH-H_2_O, 60% MeOH-H_2_O, 80% MeOH-H_2_O, and 100% MeOH) to yield six fractions (EA0, EA2, EA4, EA6, EA8, and EA10). Fraction EA4 (3.8 g) was further applied to silica gel column chromatography (180 g, CH_2_Cl_2_-MeOH (20:1→1:1) gradient solvent system) to give seven fractions (EA4A-EA4G). On the basis of the LC/MS results, fraction EA4C (1.5 g) was separated using RP-C18 column chromatography with the MeOH-H_2_O solvent system (10–100% MeOH) to obtain seven subfractions (EA4C1-EA4C7). Fraction EA4C4 (0.8 g), which was found to have major phenolic compounds, as determined using the LC/MS analysis, was subjected to preparative reversed-phase HPLC with the MeOH-H_2_O solvent system (10–80% MeOH) to obtain four fractions (EA4C41-EA4C44). Finally, fraction EA4C42 (174.5 mg) was purified by semipreparative HPLC (18% MeOH-H_2_O) to give compounds **1** (retention time (*t*_R_) 35.5 min, 21.4 mg), **2** (*t*_R_ 42.0 min, 43.9 mg), and **3** (*t*_R_ 23.2 min, 31.8 mg).

### 2.3. Cell Culture and Treatment

Normal human dermal fibroblast (NHDF) was purchased from PromoCell GmbH (Sickingenstr, Heidelberg, Germany). The cells were cultured in Dulbecco’s Modified Eagle Medium (DMEM; Corning, Manassas, VA, USA) containing 10% fetal bovine serum (FBS; Atlas, Fort Collins, CO, USA) and 1% penicillin–streptomycin solution (Gibco, Grand Island, NY, USA) in a humid atmosphere at 5% of CO_2_ and 37 °C. Dimethyl sulfoxide (DMSO; Sigma-Aldrich, St. Louis, MO, USA) was used as vehicle of compounds **1**–**3**, and kept under 0.1% final percentage. TNF-α (PeproTech, Rock Hill, NJ, USA) was prepared by dissolving in autoclaved distilled water. In each cell experiment, cells were seeded and incubated for 24 h, and then it was starved with serum-free DMEM for 24 h. After that, each compound and TNF-α were treated.

### 2.4. Assessment of Intracellular ROS

The NHDFs were seeded in a 96-well black plate at density 1 × 10^4^ cells/well and incubated for 24 h. After starvation for 24 h, the cells were treated with 50 and 100 µM compound **1** for 1 h, and then with 20 ng/mL TNF-α for 15 min. Subsequently, the cells were stained with 10 µM dichlorofluorescein diacetate (DCFDA; Sigma-Aldrich) for 15 min and washed with phosphate-buffered saline (PBS; Welgene, Gyeongsangbuk, Korea). The fluorescence was measured at wavelengths of 485/535 nm using SPARK 10M (Tecan Group Ltd., Männedorf, Switzerland). ROS accumulation was calculated based on a ratio to 100% of the vehicle control (DMSO). Fluorescent pictures were observed using a fluorescence microscope IX51 (Olympus, Tokyo, Japan).

### 2.5. Real-Time Reverse Transcription PCR (qRT-PCR)

Isolation and purification of total RNA were performed using the RNeasy Mini Kit (Qiagen, Germantown, MD, USA). cDNA synthesis was performed using the RevertAid First Strand cDNA synthesis kit (Thermo Fisher Scientific, Eugene, Oregon, USA). qPCR was performed using the QuantStudio™ 3 Real-Time PCR System (Applied Biosystems, Waltham, CA, USA) and PowerUp SYBR PCR Master Mix (Applied Biosystems, Waltham, CA, USA). PCR primers were as follows: matrix metalloproteinase-1 (MMP-1), 5′-ATTCTACTGATATCGGGGCTTT-3′, and 5′-ATGTCCTTGGGGTATCCGTGTA-3′; procollagen I α1 (COLIA1), 5′-CTCGAGGTGGACACCACCCT-3′, and 5′-CAGCTGGATGGCCACATCGG-3′; interleukin-1β (IL-1β), 5′-CTGTCCTGCGTGTTGAAAGA-3′, and 5′-TTCTGCTTGAGAGGTGCTGA-3′; interleukin-6 (IL-6), 5′-CTCCTTCTCCACAAGCGCC-3′, and 5′-GCCGAAGAGCCCTCAGGC-3′; and β-actin, 5′-AGAGATGGCCACGGCTGCTT-3′, and 5′-ATTTGCGGTGGACGATGGAG-3′. After preheating at 95 °C, the amplifications were performed by 40 cycles at 95 °C for 1 s, 60 °C for 30 s. Relative gene expression levels were normalized with β-actin and calculated based on a ratio to 100% of the vehicle control (DMSO).

### 2.6. Enzyme-Linked Immunosorbent Assay (ELISA)

The NHDFs were seeded in a 48-well plate at density 4 × 10^4^ cells/well and incubated for 24 h. After starvation for 24 h, the cells were treated with 50 and 100 µM compound **1** for 1 h, and then with 20 ng/mL TNF-α for 12 h (IL-1β and IL-6) and 24 h (MMP-1 and COLIA1). Subsequently, the supernatant was collected and the concentration of IL-1β, IL-6, MMP-1, and COLIA1 was measured using a sandwich ELISA kit (R&D systems, Minneapolis, USA).

### 2.7. Western Blotting

The NHDFs were seeded in a 6-well plate at density 2 × 10^5^ cells/well and incubated for 24 h. After starvation for 24 h, the cells were treated with 50 and 100 µM compound **1** for 1 h, and then with 20 ng/mL TNF-α for 15 min and 6 h. The cells were washed with PBS and lysed with Radioimmunoprecipitation (RIPA) buffer (Tech & Innovation, Gangwon, Korea) containing phosphatase inhibitor cocktail 2 and 3 (Sigma-Aldrich, St. Louis, MO, USA) and protease inhibitor cocktail (Roche Diagnostics, Indianapolis, IN, USA). The cell lysates were centrifuged at 13,000 rpm for 20 min at 4 °C, and the supernatant was collected. The protein was mixed with 4× Laemmli sample buffer (Bio-Rad Laboratories; Inc., Hercules, CA 94547, USA) and boiled for 10 min. The protein samples were separated on a sodium dodecyl sulfate polyacrylamide gel (SDS-PAGE) by electrophoresis and transferred onto polyvinylidene difluoride membranes (PVDF; Merck Millipore, Darmstadt, Germany). Subsequently, the membranes were blocked with 5% skim milk and then washed with Tris-buffered saline containing 0.1% Tween-20 (TBS-T). The membranes were incubated overnight at 4 ℃ with primary antibodies (extracellular-signal-regulated kinas (ERK) 1/2, phospho-ERK1/2, p38, phospho-p38, c-Jun N-terminal kinase (JNK), phospho-JNK, protein kinase B (Akt), phospho-Akt, heme oxygenase-1 (HO-1), cyclooxygenase-2 (COX-2), and glyceraldehyde 3-phosphate dehydrogenase (GAPDH); Cell Signaling, Danvers, MA, USA). After washing with TBS-T, the membranes were incubated with respective horseradish–peroxidase-conjugated secondary antibodies (Santa Cruz Biotechnology, CA, USA) for 1 h. After washing with TBS-T, the immunoreactive bands were visualized by Fusion Solo Chemiluminescence System (PEQLAB Biotechnologie GmbH, Erlangen, Germany) and SuperSignal^®^ West Femto Maximum Sensitivity Chemiluminescent Substrate (Thermo Fisher Scientific, Rockford, IL, USA). Band densities were calculated using Image J software (Version 1.51J, National Institutes of Health, Bethesda, MD, USA).

### 2.8. Statistical Analysis

The results are presented as mean ± SEM. Statistical differences were carried out by a one-way analysis of variance (ANOVA) and Tukey’s post-hoc test. It was considered statistically significant when *p* < 0.05 as compared to non-treated cells.

## 3. Results and Discussion

### 3.1. Isolation and Structural Identification of Compounds

**Figure 1:**
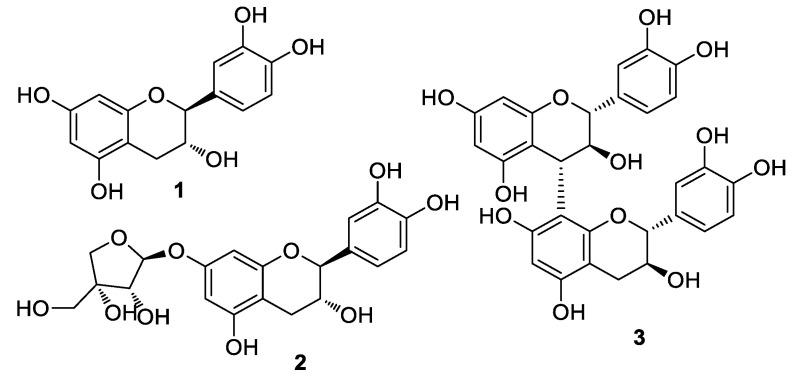
Structure of compounds **1**–**3**.

The crude extract of *U. davidiana* root bark was solvent-partitioned between water and organic solvents (hexane, CH_2_Cl_2_, EtOAc, and *n*-BuOH) of increasing polarity, yielding four fractions. By comprehensive LC/MS, the EtOAc-soluble fraction was selected for further chemical analysis of phenolic compounds and isolated compounds **1**–**3** (Figure 1). The isolated compounds were structurally elucidated as (-)-catechin (**1**) [26], (-)-catechin-7-*O*-β-d-apiofuranoside (**2**) [27], and procyanidin B3 (**3**) [28,29], based on the spectroscopic data, including 1D and 2D NMR and LC/MS analyses (Figures S1–S4). Their absolute configurations were established by comparing the ECD data and specific rotation.

Tea catechins are among the popular phenolic compounds and found in various plants [30]. Catechins are strong antioxidants, but some catechins can act as a pro-oxidant in cells. As a pro-oxidant, they can cause cell death by increasing ROS generation [31,32]. Structural differences in catechins are considered important for antioxidative activity. Among the catechins, (-)-epigallocatechin gallate and (+)-catechin have been reported to be beneficial in preventing and protecting against diseases caused by oxidative stress [30]. As our preliminary analysis showed that (-)-catechin (**1**) has antioxidative effect without cytotoxicity (Figures S5 and S6), we considered that it might ameliorate skin aging associated with oxidative stress. However, the anti-skin aging effect of (-)-catechin under oxidative stress has not been reported. Thus, in this study, we focused on (-)-catechin (**1**) among the catechins isolated from root bark of *U. davidiana*.

### 3.2. Effect of (-)-Catechin on MMP-1 and Procollagen I α1 mRNA and Protein Expression in TNF-α-Stimulated NHDFs

UV exposure results in the accumulation of free radical species (ROS), which alter gene and protein structure and function in the skin. It also increases the activity of collagenases such as MMP-1 and degrades collagen in the skin extracellular matrix, leading to wrinkle formation in the skin [8,33]. Therefore, inhibitors of collagenase activity are considered potential treatments for skin aging, including wrinkle formation [34].

**Figure 2:**
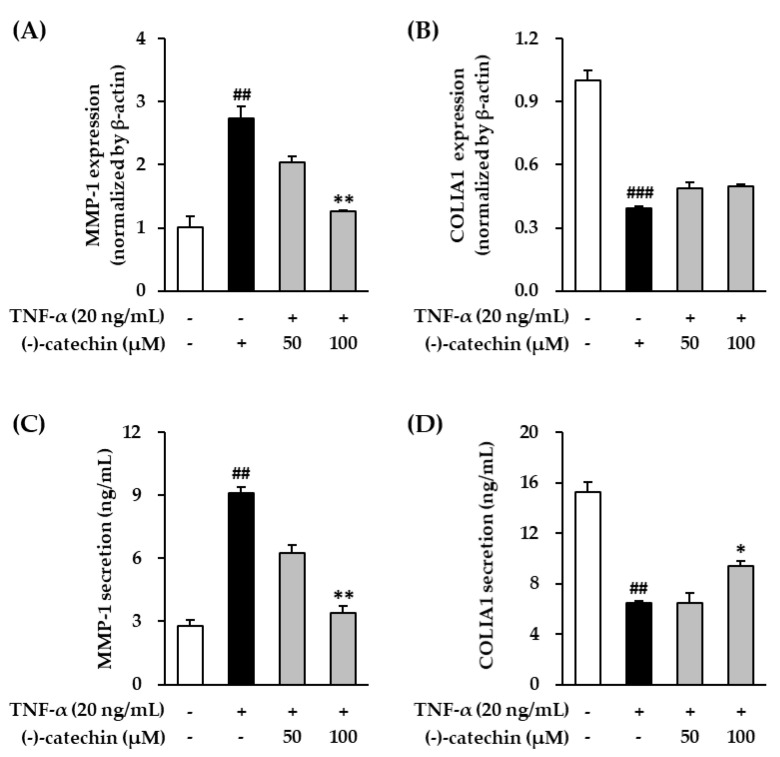
Effect of (-)-catechin on *MMP-1* and *COLIA1* mRNA and protein expression in tumor necrosis factor-α (TNF-α)-stimulated normal human dermal fibroblasts (NHDFs). (**A**,**B**) The NHDFs were treated with 50 and 100 µM (-)-catechin for 1 h, and then with 20 ng/mL TNF-α for 4 h. The matrix metalloproteinase-1 (*MMP-1*) and procollagen I α1 *(COLIA1*) mRNA levels were measured by qRT-PCR. (**C**,**D**) The NHDFs were treated with 50 and 100 µM (-)-catechin for 1 h, and then with 20 ng/mL TNF-α for 12 h. The MMP-1 and COLIA1 levels were measured using an ELISA kit. The data are presented as mean ± SEM (N = 3). ## *p* < 0.01 and ### *p* < 0.001 compared with the untreated group; * *p* < 0.05 and ** *p* < 0.01 compared with the TNF-α-treated group.

Tumor necrosis factor-α can mediate the harmful effects of UV radiations, including UVB; it is secreted from skin fibroblasts and keratinocytes and plays an important role in photoaging [35]. We preferentially investigated the inhibitory effect of (-)-catechin on MMP-1 mRNA and protein expression in TNF-α-stimulated NHDFs. The cells were treated with (-)-catechin, and then stimulated with TNF-α. As shown in Figure 2A, the stimulation with TNF-α increased MMP-1 mRNA expression; however, its expression was decreased by (-)-catechin in a concentration-dependent manner. Similarly, increased MMP-1 protein expression by TNF-α stimulation was also significantly reversed by (-)-catechin treatment (*p* < 0.05) (Figure 2C). MMP-1 is a collagenase that cleaves procollagen I α1 (COLIA1), which is the most abundant structural protein in the skin. Thus, we measured COLIA1 mRNA expression. As shown in Figure 2B, TNF-α suppressed COLIA1 mRNA expression; however, it was not recovered by (-)-catechin treatment; whereas, the reduced COLIA1 expression by TNF-α was significantly reversed by (-)-catechin treatment (*p* < 0.05) (Figure 2D). These results showed that MMP-1 was suppressed by (-)-catechin in TNF-α-stimulated NHDFs, and it prevented the degradation of COLIA1. A previous study reported that (-)-epigallocatechin gallate, which is a structure conjugated with gallate, has a protective effect against collagenase activity by regulating the nuclear factor kappa B (NF-κB), Activator protein 1 (AP-1), and MAPK signaling pathways [30]. Similarly, (-)-catechin is considered an inhibitor of collagenase, and it may prevent wrinkles caused by the damage of skin extracellular matrix.

### 3.3. Inhibitory Effect of (-)-Catechin on Intracellular ROS Production in TNF-α-Stimulated NHDFs

**Figure 3:**
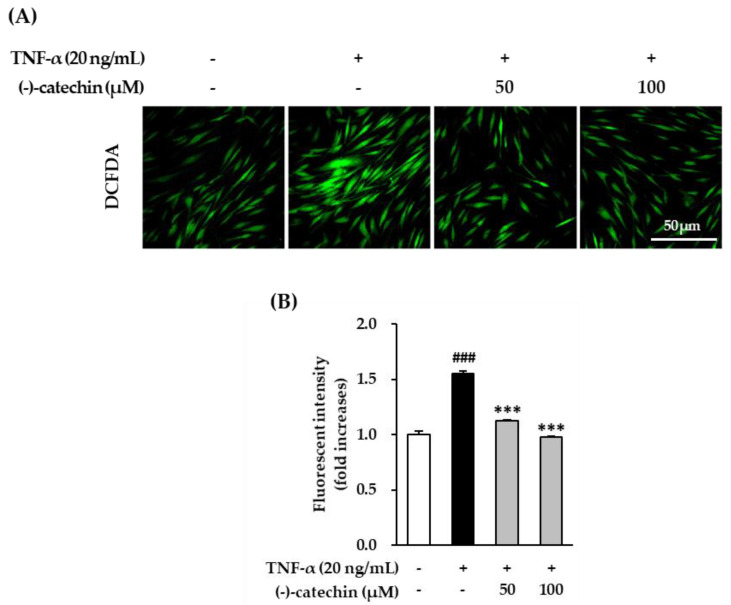
Inhibitory effect of (-)-catechin on intracellular reactive oxygen species (ROS) production in tumor necrosis factor-α (TNF-α)-stimulated normal human dermal fibroblasts (NHDFs). The cells were seeded at a density of 1 × 10^4^ in a 96-well plate and incubated for 24 h. The cells were continuously starved in serum-free Dulbecco’s Modified Eagle Medium (DMEM) for 24 h. Thereafter, the cells were treated with 50 and 100 µM (-)-catechin for 1 h, and then with 20 ng/mL TNF-α and 10 µM dichlorofluorescin diacetate (DCFDA) for 15 min. (**A**) Fluorescent images were captured by fluorescence microscopy (scale bar = 50 µm). (**B**) The graph presents the fold-increase in fluorescent intensity compared with the untreated group. The data are presented as mean ± SEM (N = 3). ### *p* < 0.001 compared with the untreated group; *** *p* < 0.001 compared with the TNF-α-treated group.

As described above, ROS accumulation causes skin damage via the degradation of collagen in the skin extracellular matrix. To evaluate the antioxidative effect of (-)-catechin, we assessed the inhibitory effect of intracellular ROS production by (-)-catechin in TNF-α-stimulated NHDFs. To measure intracellular ROS production, the cells were stained with fluorogenic dye dichlorofluorescein diacetate (DCFDA). Starved NHDFs were treated with (-)-catechin and subsequently with TNF-α and 10 µM DCFDA for 15 min, and then fluorescent images were captured. The TNF-α-stimulated cells showed strong green fluorescence (Figure 3A), indicating ROS accumulation. This increased ROS generation was inhibited by (-)-catechin treatment. The graph in Figure 3B shows that fluorescent intensity in the TNF-α-stimulated group was increased by 1.55 ± 0.03-fold compared with that in the non-treated group. Furthermore, it was substantially reduced to 1.12 ± 0.01- and 0.97 ± 0.01-fold (*p* < 0.001) after treatment with 50 and 100 µM (-)-catechin, respectively. These results showed that ROS accumulation was suppressed in TNF-α-stimulated NHDFs by (-)-catechin, and this might be the mechanism involved in ameliorating skin aging under oxidative stress.

### 3.4. Effect of (-)-Catechin on TNF-α-Induced Phosphorylation of MAPKs in NHDFs

**Figure 4:**
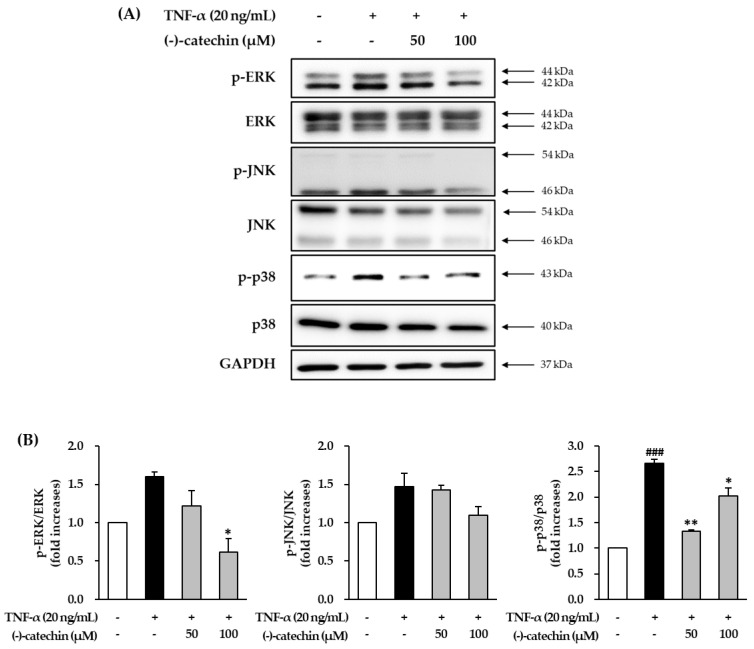
Effect of (-)-catechin on tumor necrosis factor-α (TNF-α)-induced phosphorylation of mitogen-activated protein kinases (MAPKs) in normal human dermal fibroblasts (NHDFs). (**A**) The cells were treated with 50 and 100 µM (-)-catechin for 1 h, and then with 20 ng/mL TNF-α for 15 min. The immunoreactive bands were analyzed by immunoblotting of p-JNK, JNK, p-ERK, ERK, p-p38, p38, and GAPDH. (**B**) The graphs present the fold-increase in the phosphorylation of MAPKs compared with the untreated group. The data are presented as mean ± SEM (N = 3). ### *p* < 0.001 compared with the untreated group; * *p* < 0.05 and ** *p* < 0.01 compared with the TNF-α-treated group.

To investigate the mechanism underlying the anti-skin aging effect of (-)-catechin, we investigated the effect on TNF-α-induced phosphorylation of MAPKs in NHDFs. The starved NHDFs were treated with (-)-catechin, and then with TNF-α for 15 min; thereafter, the cells were subjected to Western blotting. The TNF-α-stimulated cells showed high phosphorylation of ERK, JNK, and p38 (Figure 4A), and it was reduced by (-)-catechin treatment. The ratio of phospho-ERK (p-ERK)/ERK in the TNF-α-stimulated group was 1.60 ± 0.06-fold higher than that in the non-treated group (Figure 4B). Furthermore, it was significantly decreased to 1.22 ± 0.20- and 0.97 ± 0.17-fold (*p* < 0.05) by treatment with 50 and 100 µM (-)-catechin (Figure 4B). Similarly, the phosphorylation of p38 was substantially increased in the TNF-α-stimulated group to 2.67 ± 0.08-fold compared with the non-treated group, and (-)-catechin reduced it to 1.34 ± 0.02- and 2.02 ± 0.16-fold (*p* < 0.05) after treatment with 50 and 100 µM (-)-catechin. Because the decrease was not concentration-dependent, and high concentrations of (-)-catechin may be considered to increase phosphorylation of p38. The phosphorylation of JNK was also increased in the TNF-α-stimulated group compared with the non-treated group, but the (-)-catechin-treated group showed a decreasing tendency. These results showed that (-)-catechin suppresses TNF-α-induced phosphorylation of MAPKs.

Studies have reported that antioxidants inhibit ROS accumulation, and thus prevent MAPK activation. This indicates that ROS promote the activation in MAPK signaling [36,37,38]. The MAPKs including p38, ERK, and JNK have important functions in increasing the activity of collagenases such as MMP-1 [39,40]. Tumor necrosis factor-α-induced ROS accumulation causes the degradation of collagen in the skin extracellular matrix via MAPK activation [41]. Overall, our results showed that (-)-catechin may prevent MMP-1 synthesis by inhibiting MAPK activation.

### 3.5. Effect of (-)-Catechin on the Phosphorylation of Akt and Expression of COX-2 and HO-1 in TNF-α-Stimulated NHDFs

**Figure 5:**
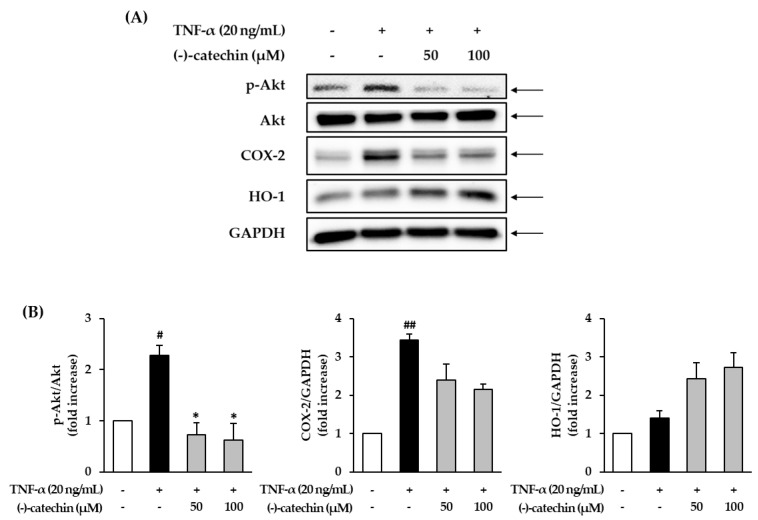
Effect of (-)-catechin on the phosphorylation of Akt and expression of cyclooxygenase-2 (COX-2) and heme oxygenase-1 (HO-1) in tumor necrosis factor-α (TNF-α)-stimulated normal human dermal fibroblasts (NHDFs). (**A**) The cells were treated with 50 and 100 µM (-)-catechin for 1 h, and then with 20 ng/mL TNF-α for 6 h. The immunoreactive bands of the immunoblotting analysis of p-Akt, Akt, COX-2, HO-1, and GAPDH. (**B**) The graph presents the fold-increase in the phosphorylation of Akt and expression of COX-2 and HO-1 compared with the untreated group. The data are presented as mean ± SEM (N = 3). # *p* < 0.05 and ## *p* < 0.01 compared with the untreated group; * *p* < 0.05 compared with the TNF-α-treated group.

Excessive accumulation of ROS results in the activation of Akt and upregulates the production of nuclear factor kappa B (NF-κB) and cyclooxygenase-2 (COX-2), causing inflammation [42,43]. Thus, we investigated the effect of (-)-catechin on the phosphorylation of Akt and expression of COX-2 in TNF-α-stimulated NHDFs. The starved NHDFs were treated with (-)-catechin, and then with TNF-α for 6 h; thereafter, the cells were subjected to Western blotting. The TNF-α-stimulated group showed increased Akt phosphorylation and COX-2 expression compared with the non-treated group (Figure 5A), and this was inhibited by (-)-catechin treatment. The ratio of phospho-Akt (p-Akt)/Akt in the TNF-α-stimulated group was considerably increased to 2.28 ± 0.19-fold (*p* < 0.05) compared with the non-treated group (Figure 5B); however, it was apparently reduced by (-)-catechin treatment. Analogously, COX-2 expression was increased by TNF-α stimulation, and it was decreased by (-)-catechin treatment. Previous studies have demonstrated that phenolic compounds, including catechins, inhibit inflammatory mediators (NO and COX-2) and suppress inflammatory responses via the NF-κB pathway [44,45]. Similar to these findings, in the present study, (-)-catechin activated the NF-κB pathway by suppressing the inflammatory mediators such as COX-2. Thus, (-)-catechin may ameliorate inflammation induced by ROS accumulation.

Studies have reported that heme oxygenase-1 (HO-1) can inhibit free radical generation, and thus prevent inflammatory damage and apoptosis of human skin cells [46,47]. In the present study, the expression of HO-1 did not show a significant change with TNF-α stimulation, but it was significantly increased by (-)-catechin at 50 and 100 µM concentrations to 2.43 ± 0.41- and 2.73 ± 0.38-fold (*p* < 0.05), respectively. This result suggests that (-)-catechin may prevent ROS accumulation induced by TNF-α stimulation by capturing free radicals by HO-1.

### 3.6. Effect of (-)-Catechin on Proinflammatory Cytokines in TNF-α-Stimulated NHDFs

**Figure 6:**
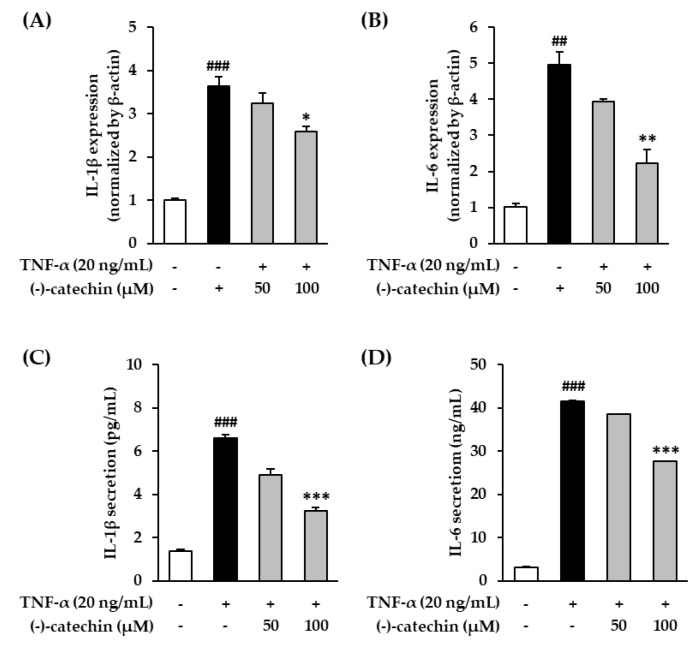
Effect of (-)-catechin on proinflammatory cytokines interleukin (IL)-1β and IL-6 in tumor necrosis factor-α (TNF-α)-stimulated normal human dermal fibroblasts (NHDFs). (**A**,**B**) The cells were treated with 50 and 100 µM (-)-catechin for 1 h, and then with 20 ng/mL TNF-α for 4 h. The mRNA level of *IL-1β* and *IL-6* was measured by qRT-PCR. (**C**,**D**) The NHDFs were treated with 50 and 100 µM (-)-catechin for 1 h, and then with 20 ng/mL TNF-α for 12 h. The levels of IL-1β and IL-6 were measured using the ELISA kit. The data are presented as mean ± SEM (N = 3). ## *p* < 0.01 and ### *p* < 0.001 compared with the untreated group; * *p* < 0.05, ** *p* < 0.01, and *** *p* < 0.001 compared with the TNF-α-treated group.

Cellular oxidative stress is associated with proinflammatory cytokines such as TNF-α, IL-1β, and IL-6, and they upregulate inflammatory reactions [48,49]. Furthermore, they promote aging-related reactions, including skin aging [41,50]. Among these cytokines, TNF-α plays an important role in photoaging [35]. To investigate the inhibitory effect of (-)-catechin on aging-related inflammatory reaction, we investigated the effect of (-)-catechin on *IL-1β* and *IL-6* mRNA expression in TNF-α-stimulated NHDFs. The starved NHDF cells were treated with (-)-catechin, and then with TNF-α for 4 h; thereafter, the cells were subjected to qRT-PCR. As shown in Figure 6A, the stimulation with TNF-α increased *IL-1β* mRNA expression by 3.64 ± 0.21-fold compared with that in the non-treated cells, and it was decreased by (-)-catechin at 50 and 100 µM concentration to 3.25 ± 0.24- and 2.59 ± 0.11-fold, respectively. *IL-6* mRNA expression was substantially increased to 4.96 ± 0.34-fold by TNF-α stimulation compared with that in the non-treated cells, and it was significantly reversed by (-)-catechin in a concentration-dependent manner (100 μM; 2.23 ± 0.38-fold, *p* < 0.01) (Figure 6B). To measure the expression of IL-1β and IL-6, we performed the ELISA. Consistent with the results of mRNA expression, IL-1β (from 1.36 ± 0.09 to 6.58 ± 0.18 pg/mL, *p* < 0.001) and IL-6 (from 3.14 ± 0.21 to 41.56 ± 0.32 ng/mL, *p* < 0.001) expression was apparently increased by TNF-α stimulation. The expression of both IL-1β and IL-6 was significantly reduced in a concentration-dependent manner (*p* < 0.001) (Figure 6C,D). The results suggest that (-)-catechin may prevent skin aging-related reactions induced by TNF-α stimulation via the suppression of proinflammatory cytokines.

In summary, (-)-catechin isolated from *U. davidiana* extract exerted an antioxidant effect by inhibition of intracellular ROS accumulation in TNF-α-stimulated NHDFs. (-)-Catechin prevented the degradation of the skin extracellular matrix, including increase of collagenase MMP-1 and decrease of collagen synthesis. Mechanistically, it acts via the suppression of MAPK, Akt, and COX-2 activation. Furthermore, (-)-catechin prevents TNF-α-induced ROS accumulation by capturing free radicals by HO-1. It also suppresses proinflammatory cytokines, including interleukin (IL)-1β and IL-6, which upregulate inflammatory reactions and promote aging-related reactions, including skin aging. Although the study was performed under TNF-α stimulation, which occurs ROS accumulation, (-)-catechin is expected to also protect further upstream such as with photoaging. Therefore, it is necessary to further investigate whether (-)-catechin has a protective effect on extrinsic skin aging with harmful direct UV exposure.

## 4. Conclusions

Our study demonstrated that (-)-catechin significantly suppressed the TNF-α-induced activity of MMP-1 and prevented the inhibition of collagens synthesis. The mechanism underlying the anti-skin aging effect of (-)-catechin involved the suppression of TNF-α-induced ROS accumulation and MAPK, Akt, and COX-2 activation. Furthermore, (-)-catechin suppressed the expression of TNF-α-induced proinflammatory cytokines, including IL-1 and IL-6. Overall, (-)-catechin is considered a therapeutic candidate for improving both intrinsic and extrinsic skin aging, because it inhibited ROS accumulation in NHDFs.

## Supplementary Materials

The following are available online at https://www.mdpi.com/2076-3921/9/10/981/s1, Figure S1: LC/MS analysis of the EtOAc fraction of *U. davidiana* var. *japonica* extract (detection wavelength was set at 254 nm) and UV spectra of compounds **1**–**3**; Figure S2: The 1H NMR spectrum of compound **1** in CD_3_OD and negative ion-mode ESI-MS data of 1; Figure S3: The 1H NMR spectrum of compound **2** in CD_3_OD and negative ion-mode ESI-MS data of 2; Figure S4: The 1H NMR spectrum of compound **3** in CD_3_OD and negative ion-mode ESI-MS data of **3**; Figure S5: Antioxidant activity of isolated compounds **1**–**3**; Figure S6: Cell viability of isolated compounds **1**–**3** against normal human fibroblast (NHDF) cells.
