# Supplementary material for: Potential Anti-Skin Aging Effect of (-)-Catechin Isolated from the Root Bark of Ulmus davidiana var. japonica in Tumor Necrosis Factor-α-Stimulated Normal Human Dermal Fibroblasts
Source: Antioxidants (Basel). 2020 Oct 13;9(10):981. doi: 10.3390/antiox9100981 (PMC7601800; doi:10.3390/antiox9100981)
Supplement: Supplementary file 1 [file antioxidants-09-00981-s001.pdf]

antioxidants

Supplementary Materials

Potential Anti-Skin Aging Effect of (-)-Catechin Isolated from the Root Bark of *Ulmus davidiana* var. *japonica* in Tumor Necrosis Factor-α-Stimulated Normal Human Dermal Fibroblasts

Sullim Lee[1], Jae Sik Yu[2], Hung Manh Phung[3], Jeong Gun Lee[4], Ki Hyun Kim[2],* and Ki Sung Kang [3,*]

[1] Department of Life Science, College of Bio-Nano Technology, Gachon University, Seongnam 13120, Republic of Korea; sullimlee@gachon.ac.kr (S.L.)

[2] School of Pharmacy, Sungkyunkwan University, Suwon 16419, Republic of Korea; jsyu@bu.edu (J.S.Y.); khkim83@skku.edu (K.H.K)

[3] College of Korean Medicine, Gachon University, Seongnam 13120, Republic of Korea; 201940218@gc.gachon.ac.kr (H.M.P.); kkang@gachon.ac.kr (K.S.K.)

[4] S-Skin Co., Ltd., #220, 17, Daehak 4-ro, Suwon 16226, Republic of Korea; bio.lee@s-skin.com (J.G.L.)

* Correspondence: Tel.: 82-31-290-7700; E-mail: khkim83@skku.edu (K.H.K), Tel.: 82-31-750-5402; Fax: 82-31750-5416; E-mail: kkang@gachon.ac.kr (K.S.K.)

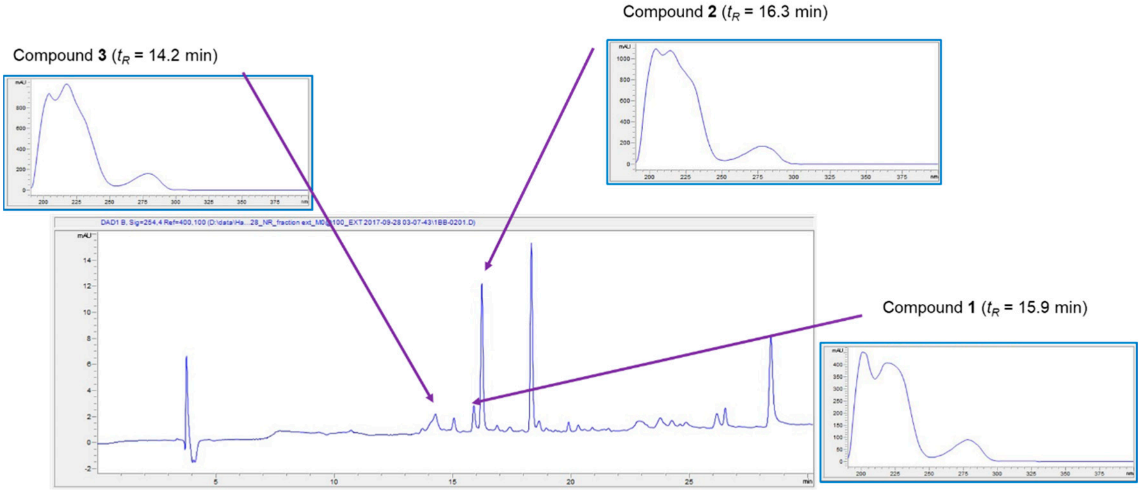

Figure S1. LC/MS analysis of the EtOAc fraction of *U. davidiana* var. *japonica* extract (detection wavelength was set at 254 nm) and UV spectra of compounds **1-3**; LC/MS analysis was performed on an Agilent 1200 Series HPLC system equipped with a diode array detector and 6130 Series ESI mass spectrometer using an analytical Kinetex C_{18} 100 Å column (100 × 2.1 mm, 5 μm; flow rate: 0.3 mL/min; Phenomenex). The mobile phase was composed of 0.1% (w/v) formic acid in water (solvent A) and methanol (solvent B) in the following gradients; 0 min (A:B 90:10), 30 min (A:B 10:90), 31 min (A:B 0:100), 41 min (A:B 0:100), 41 min (A:B 90:10), and 52 min (A:B 90:10).

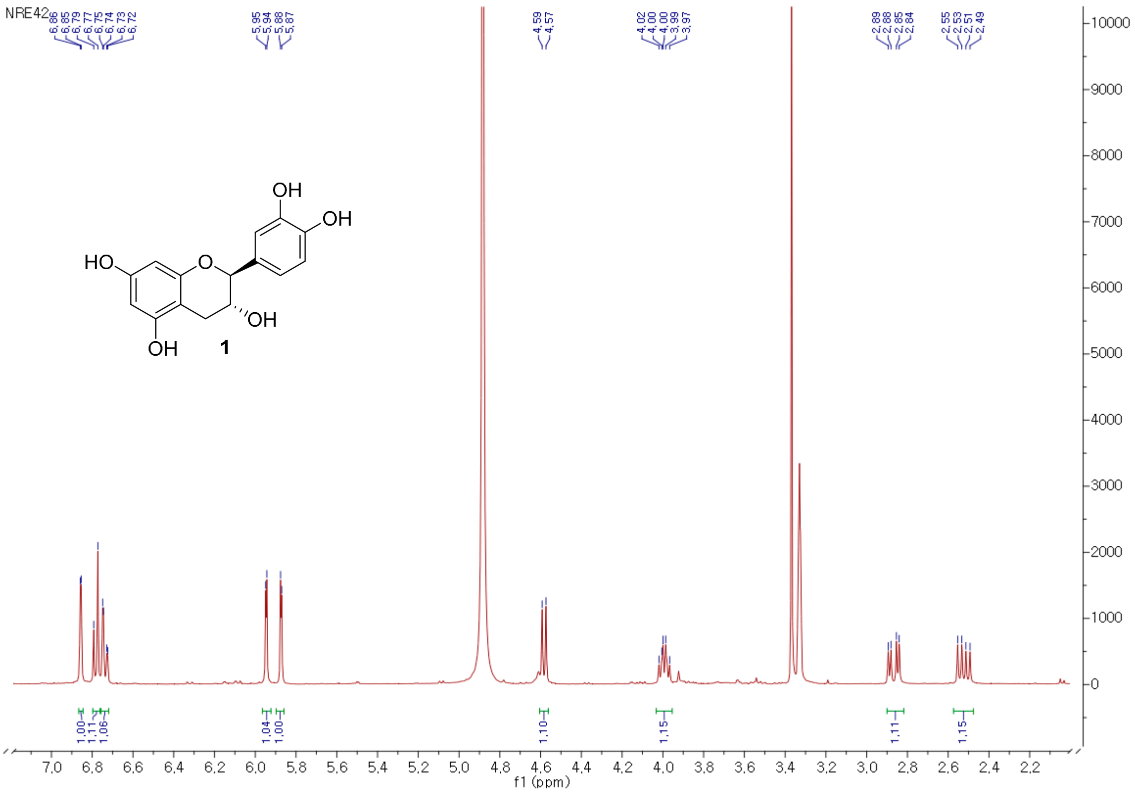

Figure S2. The 1H NMR spectrum of compound 1 in CD3OD and negative ion-mode ESI-MS data of 1.

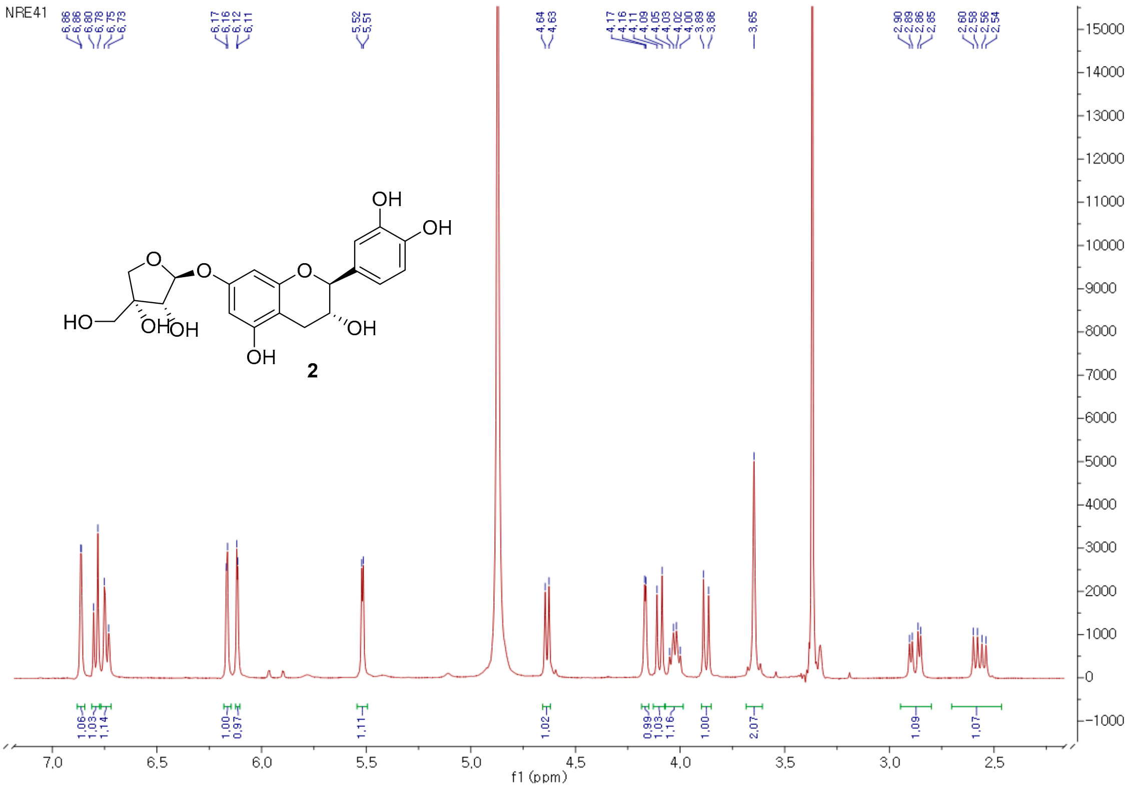

Figure S3. The ^{1}H NMR spectrum of compound **2** in CD3OD and negative ion-mode ESI-MS data of **2**.

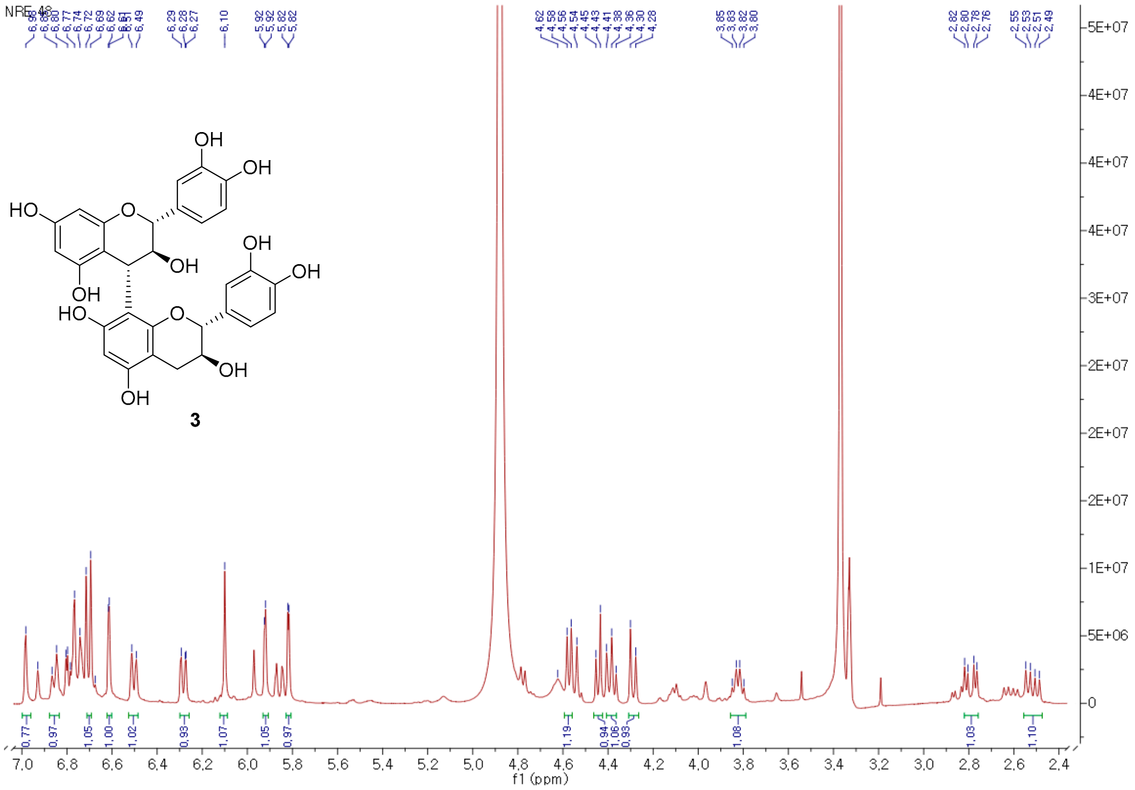

Figure S4. The ^{1}H NMR spectrum of compound **3** in CD3OD and negative ion-mode ESI-MS data of **3**.

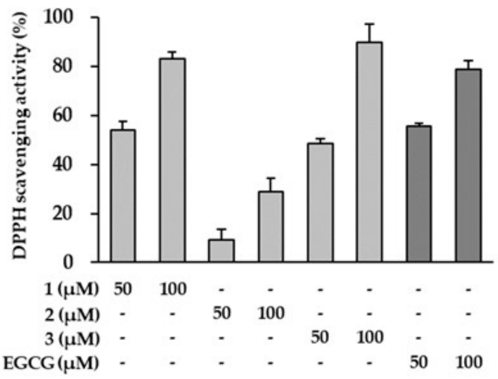

Figure S5. Antioxidant activity of isolated compounds **1** – **3**. The antioxidant activities were measured using a 1,1-diphenyl-2-picrylhydrazyl (DPPH) radical-scavenging assay. Epigallocatechin gallate (EGCG) used as positive control. The prepared compounds mixed with an equal volume of DPPH solution, and then incubated for 20 min at room temperature. The absorbances were analyzed using a microplate reader (SPARK 10M; Tecan, Männedorf, Switzerland) at wavelengths of 540 nm. Data was presented as the mean ± S.E.M (N = 3).

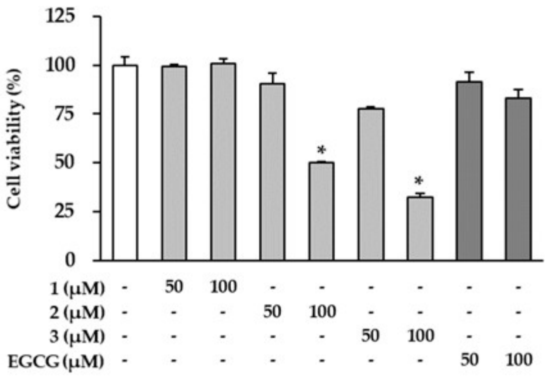

Figure S6. Cell viability of isolated compounds **1** – **3** against normal human fibroblast (NHDF) cells. The NHDF cells were exposed to the indicated compounds in a 96-well plate. Epigallocatechin gallate (EGCG) used as positive control. After 48 h, the medium in the plate was added the EZ-Cytox (Dogen, Seoul, Korea) solution. And the plate was incubated for 1 hour, the absorbances ware detected using a microplate reader (SPARK 10M; Tecan, Männedorf, Switzerland) at wavelengths of 450 nm. A cell viability of 100% was calculated from a nontreated group. Data was presented as the mean ± S.E.M (N = 3). *$p < 0.05$ compared with the nontreated group.

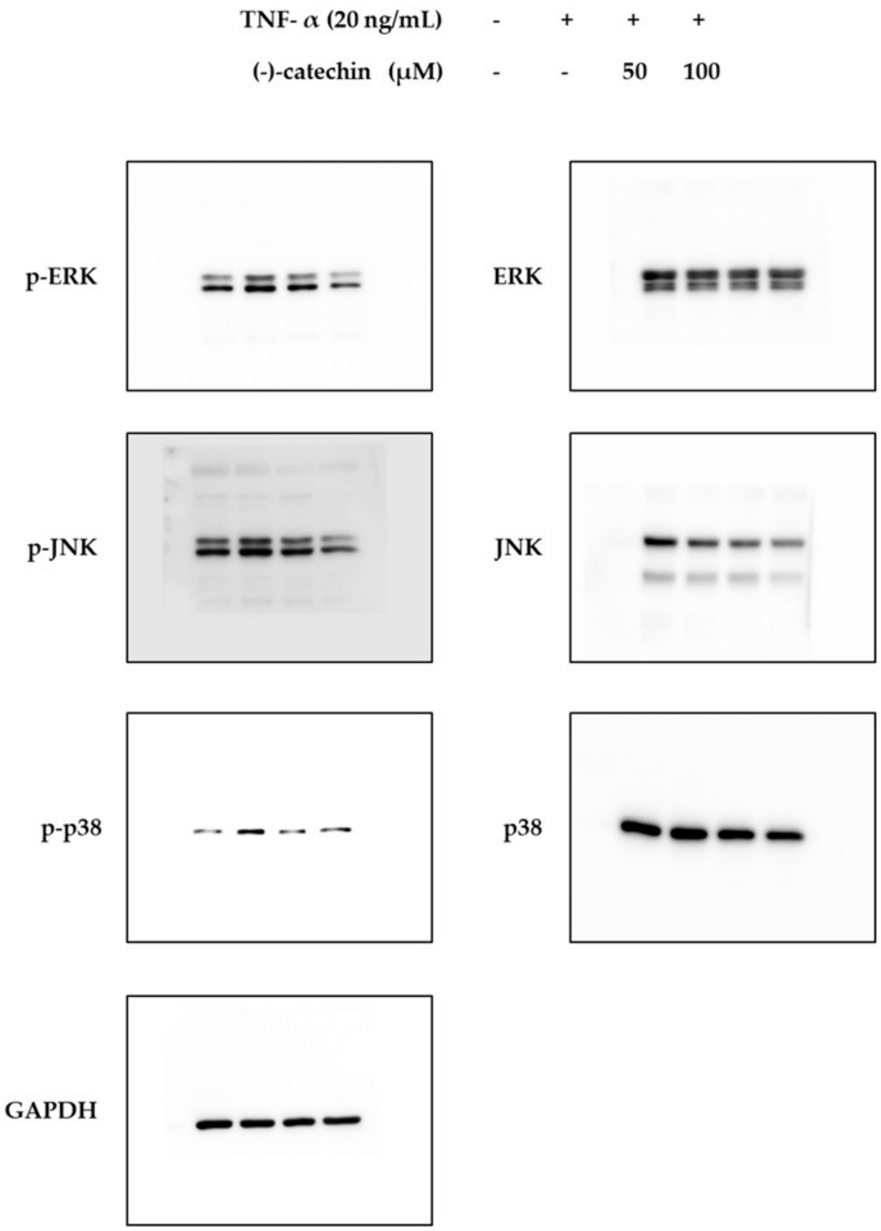

Figure S7. Effect of (-)-catechin on tumor necrosis factor-α (TNF-α)-induced phosphorylation of mitogen-activated protein kinases (MAPKs) in normal human dermal fibroblasts (NHDFs). The cells were treated with 50 and 100 μM (-)-catechin for 1 h, and then with 20 ng/mL TNF-α for 15 min. The immunoreactive bands analyzed by immunoblotting of p-JNK, JNK, p-ERK, ERK, p-p38, p38, and GAPDH.

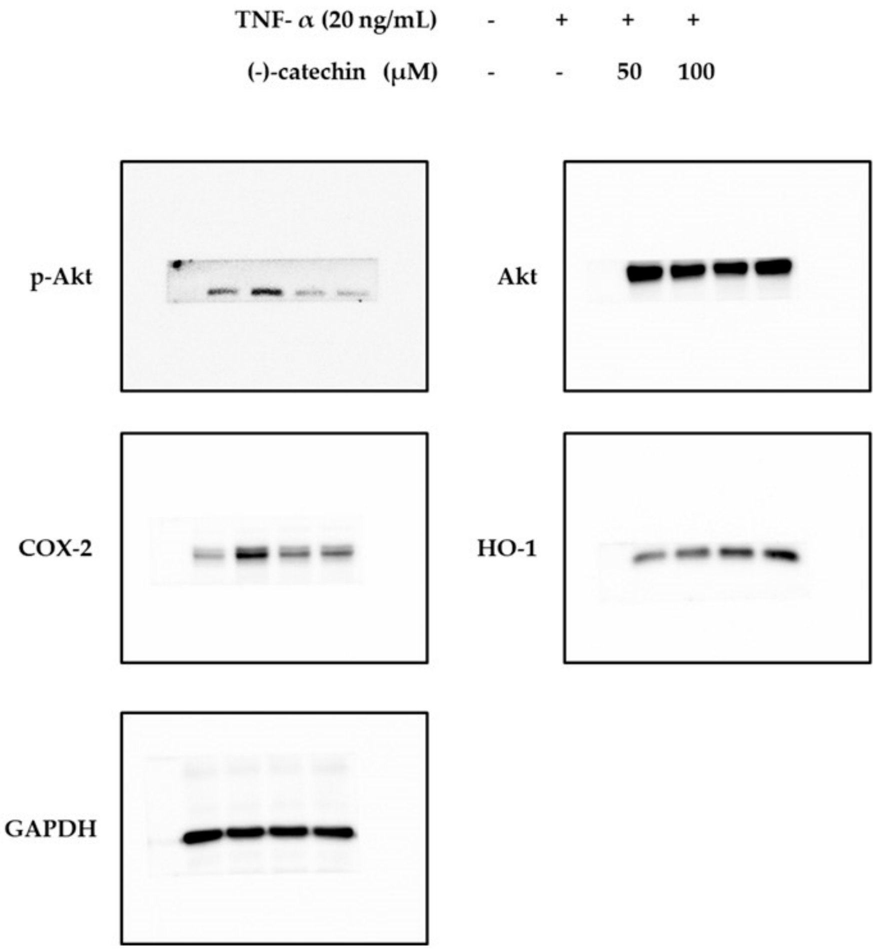

Figure S8. Effect of (-)-catechin on the phosphorylation of Akt and expression of COX-2 and HO-1 in tumor necrosis factor-α (TNF-α)-stimulated normal human dermal fibroblasts (NHDFs). The cells were treated with 50 and 100 μM (-)-catechin for 1 h, and then with 20 ng/mL TNF-α for 6 h. The immunoreactive bands of the immunoblotting analysis of p-Akt, Akt, COX-2, HO-1, and GAPDH.
